# Conditional deletion of E11/podoplanin in bone protects against load-induced osteoarthritis

**DOI:** 10.1186/s12891-019-2731-9

**Published:** 2019-07-27

**Authors:** Katherine A. Staines, Ekele Ikpegbu, Anna E. Törnqvist, Scott Dillon, Behzad Javaheri, Anish K. Amin, Dylan N. Clements, David J. Buttle, Andrew A. Pitsillides, Colin Farquharson

**Affiliations:** 1 School of Applied Sciences, Edinburgh Napier University, Sighthill Campus, Edinburgh, UK; 2 Michael Okpara University of Agriculture, Umudike, Abia State, Nigeria; 3 Rheumatology and Bone Diseases Unit, Centre for Genomic and Experimental Medicine, MRC Institute of Genetics and Molecular Medicine, University of Edinburgh, Edinburgh, UK; 4 Centre for Bone and Arthritis Research, University of Gothenburg, Gothenburg, Sweden; 5 Roslin Institute and R(D)SVS, The University of Edinburgh, Easter Bush, Midlothian, UK; 6 Comparative Biomedical Sciences, Royal Veterinary College, Royal College Street, London, UK; 7 Department of Orthopaedic and Trauma Surgery, Royal Infirmary of Edinburgh, NHS Lothian, Edinburgh, UK; 8 Department of Infection, Immunity and Cardiovascular Disease, University of Sheffield, Sheffield, UK

**Keywords:** Osteocytes, Subchondral bone, Osteoarthritis, E11/podoplanin

## Abstract

**Background:**

Subchondral bone (SCB) thickening is one of the earliest detectable changes in osteoarthritic joints and is considered a potential trigger for subsequent articular cartilage degeneration. In this manuscript, we examine whether disruption to the SCB osteocyte network contributes to the initiation and pathogenesis of osteoarthritis.

**Methods:**

We examined expression patterns of the glycoprotein E11/podoplanin by immunohistochemical labelling in murine, human and canine osteoarthritis models. We also examined the effects of twice-weekly administration of Bortezomib, a proteasome inhibitor which stabilises osteocyte E11 levels, to C57/BL6 wild-type male mice (1 mg/kg/day) for 8 weeks after surgical destabilisation of the medial meniscus. By inducing osteoarthritis-like changes in the right knee joint of 12-week-old male E11 hypomorphic mice (and corresponding controls) using a post-traumatic joint loading model, we also investigated whether a bone-specific E11 deletion in mice increases joint vulnerability to osteoarthritis. Articular cartilage degradation and osteophyte formation were assessed by histology and in line with the OARSI grading system.

**Results:**

Our studies reveal increased E11 expression in osteocytes of human and canine osteoarthritic SCB. We found that Bortezomib administration had no effect on surgically-induced osteoarthritis, potentially due to a lack of the expected stabilisation of E11 in the SCB. We also found, in concordance with our previous work, wild-type mice exhibited significant load-induced articular cartilage lesions on the lateral femoral condyle (*p* < 0.01) and osteophyte formation. In contrast, E11 hypomorphic mice did not develop osteophytes or any corresponding articular lesions.

**Conclusions:**

Overall, these data suggest that an intact osteocyte network in the SCB contributes to the development of mechanically-driven osteoarthritis. Further, the data presented here indicate that the molecular pathways that preserve the osteocyte network, such as those driven by E11, may be targeted to limit osteoarthritis pathogenesis.

**Electronic supplementary material:**

The online version of this article (10.1186/s12891-019-2731-9) contains supplementary material, which is available to authorized users.

## Background

Osteoarthritis is a degenerative joint disease and a global health care burden. In osteoarthritis, the articular cartilage undergoes structural deterioration, causing joint pain, loss of joint function and significantly reducing quality of life. However, its underlying molecular mechanisms are not fully understood. As such, there is an ever-growing need for an effective disease-modifying treatment.

Although often considered secondary, subchondral bone (SCB) thickening in osteoarthritic joints is one of the earliest detectable changes and is now considered a potential trigger for subsequent articular cartilage degeneration [1, 2]. Osteoblast-derived osteocytes are the most numerous of all the cells within bone and have a unique morphology with extensive dendritic processes creating bone’s osteocyte-canalicular network. This network is now known to orchestrate bone remodelling [3]. However, in osteoarthritic joints, the osteocytes in the SCB exhibit alterations to their exquisite dendritic morphology, with fewer and more disorganised dendrites [4]. Furthermore, other reports have noted that the expression of sclerostin, the mature osteocyte marker, is disrupted in osteoarthritic SCB [5, 6]. Together, these data suggest that the osteocyte may contribute a central role to pathological SCB sclerosis in osteoarthritis and that an intact osteocytic network is necessary for maintaining healthy SCB architecture.

Numerous genes have been suggested to influence osteocyte formation, one of which encodes the transmembrane glycoprotein E11/podoplanin. We and others have previously shown that E11 is expressed by early embedding osteocytes, thus identifying it as a factor which likely contributes to the vital, early stages of osteocyte differentiation [7–9]. It is known that mechanical strain in vivo increases E11 mRNA expression [7] and that E11 siRNA abrogates the formation of osteocyte dendrites. [7]. In contrast, over-expression of E11 in ROS 17/2.6 osteoblast-like cells has been found to promote the formation of long dendritic processes [10–12]. Furthermore, we have recently reported that E11 levels are regulated post-translationally by proteasomal degradation and that their preservation, through the administration of proteasome inhibitors such as Bortezomib, leads to the induction of an osteocyte-like morphology in MLO-A5 pre-osteocytic cells [9]. In accordance with this, we recently showed that the hypomorphic bone-specific ablation of E11 in mice results in disrupted osteocytic dendrite formation, which supports a key role for E11 in regulating the cytoskeletal changes associated with osteocyte process formation and elongation [13].

As the formation of such dendritic processes is a key functional feature of the normal mature osteocyte network, which is perturbed in osteoarthritis [4], we have examined herein whether disruption to the integrity of the osteocyte dendritic processes contributes to the initiation of osteoarthritis. Specifically, we investigated whether proteasome inhibition can stabilise E11 expression in vivo to protect against the osteoarthritis that develops following surgically-induced destabilisation of the medial meniscus (DMM). Moreover, we examined whether the bone-specific conditional deletion of E11 in mice affects early adaptive processes and joint vulnerability to osteoarthritis induction by a mechanically-induced post-traumatic osteoarthritis model.

## Methods

### Animals

C57/BL6 mice harbouring a conditional deletion of E11 in late osteoblasts (osteocalcin promoter driven; cKO; hypomorphic with ~ 70% reduction in E11 protein expression) as well as their appropriate *E11*^flox/flox^ control littermates (WT) were kept in polypropylene cages, with light/dark 12-h cycles, at 21 ± 2 °C, and fed ad libitum with maintenance diet (Special Diet Services, Witham, UK) [13]. We obtained floxed Pdpn mice from the UCOMM/KOMP, MRC Harwell, Oxfordshire, UK and osteocalcin-cre mice as a kind gift from Thomas Clemens at John Hopkins Medicine, Baltimore, Maryland. cKO and WT mice were generated as described previously [13]. All analyses were conducted blindly to minimise the effects of subjective bias. All experimental protocols were approved by Roslin Institute’s Animal Users Committee and the animals were maintained in accordance with UK Home Office guidelines for the care and use of laboratory animals.

### In vivo joint loading

12-week old male cKO (*n* = 5) and WT (*n* = 3) mice were isoflurane-anaesthetised (4% and maintained at 2% during loading) and the right knee joint loaded as described previously [14]. Briefly, using a servo-electric materials testing machine (Electroforce 3100, Bose, UK), axial compressive loads were applied through the right knee joint via custom-made cups. All studies used a single loading pattern in which peak loads of 11 N for the cKO and 12 N for the WT mice (see [13]) were applied (for 0.05 s; 0.025 s rise and fall time; 9.9 s baseline hold time at between periods of peak loading). Joints were loaded for 40 cycles, 3 times/week for 2 weeks in the morning and the left (non-loaded control) and right knees dissected 3 days after the final loading episode. Knee joints were fixed in 4% paraformaldehyde for 24 h at 4 °C before being stored in 70% ethanol. Mice were sacrificed by exsanguination and confirmation of death by cervical dislocation.

### Destabilisation of the medial meniscus (DMM)

Osteoarthritis was induced in 8-week old C57/BL6 male mice (Charles River) by surgically induced DMM under isoflurane-induced anaesthesia (see above). Animals were randomly allocated to treatment groups to reduce subjective bias. We elected not to perform sham surgery on the contralateral knee based on animal welfare grounds since previous studies had shown no difference in osteoarthritis scores between non-operated and sham-operated knee joints using this model and since the primary aim of the experimental research was to evaluate the potential protective effect of Bortezomib following DMM [15–17]. Following transection of the medial meniscotibial ligament, the joint capsule and skin were closed and anaesthesia reversed. Mice then either received twice-weekly morning intraperitoneal injections of Bortezomib (1 mg/kg [18]; *n* = 8) or vehicle control (99.7% w/v saline; n = 8) for 8 weeks at which point knee joints were dissected, fixed in 4% paraformaldehyde for 24 h at 4 °C, and then stored in 70% ethanol. Mice were sacrificed by exsanguination and confirmation of death by cervical dislocation.

### Micro-computed tomography (microCT) analysis

Scans were performed with an 1172 X-Ray microtomograph (Skyscan, Belgium) to evaluate the SCB. High-resolution scans with an isotropic voxel size of 5 μm were acquired (50 kV, 200 μA, 0.5 mm aluminium filter, 0.6° rotation angle). The projection images were reconstructed using NRecon software version 1.6.9.4 (Skyscan, Belgium). Each dataset was rotated in Dataviewer (Skysan, Belgium) to ensure similar orientation and alignment for analysis. Hand-drawn regions of interests (ROI) of the SCB trabecular bone for each femur/tibia lateral/medial compartments was first achieved [19]. SCB ROIs was subsequently selected for each compartment. Analysis of SCB plate thickness and the epiphyseal trabecular bone was achieved using 3D algorithms in CTAn (Skyscan, Belgium) to provide: SCB plate thickness (SCB Th.; mm); epiphyseal trabecular bone volume/tissue volume (Tb. BV/TV; %); trabecular number (Tb. N.; mm^− 1^); trabecular thickness (Tb. Th.; mm); trabecular separation (Tb. Sp.; mm); trabecular pattern factor (Tb. Pf.: mm^− 1^) .

### Human and animal osteoarthritic samples

The human SCB samples were obtained from patients undergoing total knee replacement for osteoarthritis. Samples (discarded femoral and tibial bone cuts) were obtained with patient consent and ethical approval from the NHS Lothian Bioresource. The collection, storage, and subsequent use of human tissues are regulated in Scotland by The Human Tissue Act (Scotland) 2006. Canine osteoarthritis samples were residual tissues collected from pets undergoing surgery for the treatment of elbow joint disease with informed consent (osteoarthritis), or which had died of unrelated disease (healthy). Consent for use was obtained from the animal owners and ethical approval for their collection and use given by the Veterinary Ethical Review Committee of the University of Edinburgh (VERC; approval 23/12). In all cases, the joints were macroscopically evaluated for signs of osteoarthritis.

### Histological analysis

Murine left and right knee joints were decalcified, wax-embedded and 7 μm coronal sections cut. For assessment of osteoarthritis severity, multiple sections (five/slide) from 120 μm intervals across the whole joint were stained with Toluidine blue (0.4% in 0.1 M acetate buffer, pH 4) and counterstained with Fast Green (0.2% in dH_2_O). Articular cartilage lesion severity was graded using the well-established OARSI grading scale [20]. Scoring was conducted blindly with a second observer scoring ~ 25% of the sections. For assessment of osteoclast activity, slides were stained with Goldner’s Trichrome using standard procedures or for tartrate resistant acid phosphatase (TRAP). For TRAP staining 70 mg napthol AS-TR phosphate (Sigma) was dissolved in 250 μl N-N dimethyl formamide (Sigma) and added to 50 ml 0.2 M sodium acetate buffer pH 5.2. 115 mg sodium tartrate dihydrate (Sigma) and 70 mg fast red salt TR (Sigma) was dissolved into this solution and slides were incubated at 37 °C for 2 h. Sections were counterstained in Meyer’s haematoxylin (Sigma), washed in distilled water and mounted in aqueous mounting medium (Vector Labs). Slides were imaged using a NanoZoomer slide scanning system (Hamamatsu) and histomorphometry performed using Bioquant Osteo (Bioquant Image Analysis Corporation).

### Immunohistochemistry

For immunohistochemical localisation of E11 and sclerostin, sections were dewaxed in xylene and rehydrated. Sections were incubated at 37 °C for 30 min in 1 mg/ml trypsin for antigen demasking. Endogenous peroxidases were blocked by treatment with 3% H_2_O_2_ in methanol (Sigma). Species-specific amino acid sequences for E11 and sclerostin were obtained and Clustal Omega was used to perform alignment and comparison of the mouse, human and dog sequences. The level of homology between amino acid sequences for two different species was expressed as mean percent identity (Additional file 1: Table S1). As a result of this and following optimisation, antibodies used were: E11 mouse samples (IgG polyclonal raised in goat; R&D systems; 1/100); E11 human and canine samples (IgG polyclonal raised in sheep; R&D systems; 1/500). Sclerostin mouse samples (IgG polyclonals raised in goat; R&D systems; 1/200); with appropriate controls [9]. The Vectastain ABC universal detection kit (Vector Laboratories, Peterborough, UK) was used according to the manufacturer’s instructions. The sections were finally dehydrated, counterstained with haematoxylin and mounted in DePeX. All sections to be compared were immunostained at the same time in order to standardise conditions and minimise any differences in antibody incubation times. Positive E11 and sclerostin staining in the articular cartilage chondrocytes and SCB osteocytes was readily identifiable and we were therefore able to semi-quantify staining intensity in these cell types between the different treatment groups.

### Statistical analysis

Statistical analysis of articular cartilage lesion grades compared loaded/DMM (right) and contra-lateral control (left) joints by paired Wilcoxon’s signed-rank test. *P* < 0.05 was considered statistically significant.

## Results

### E11 expression is increased in human and canine osteoarthritic SCB osteocytes

**Fig. 1:**
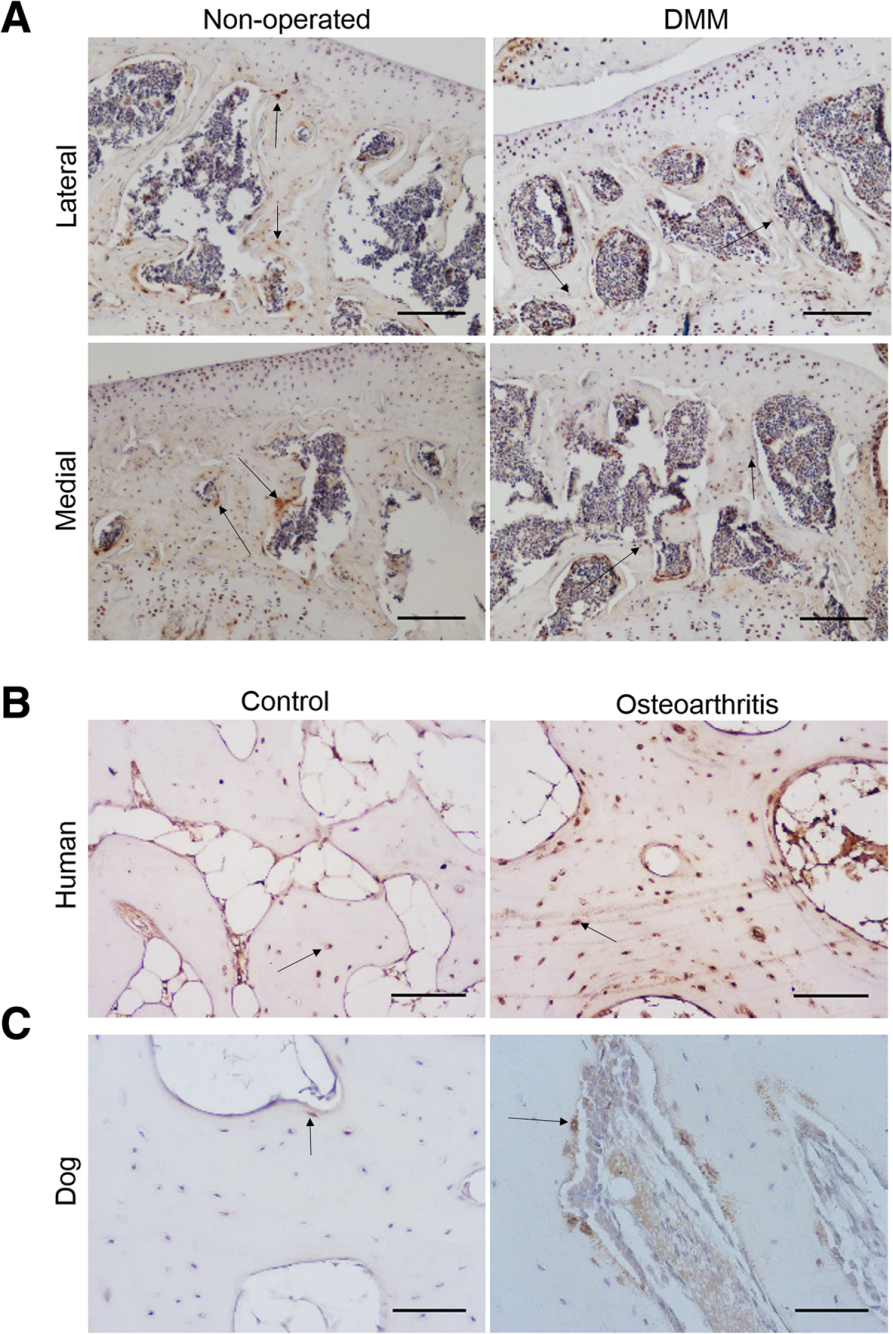
Immunohistochemical labelling for E11 in (**a**) DMM and non-operated C57/BL6 mice (**b**) human osteoarthritis samples from patients undergoing total knee replacement (**c**) canine osteoarthritis samples from the medial coronoid process of the elbow. Images are representative of *n* > 3. Arrows are representative of E11 positive osteocytes. Scale bar = 300 μm

We first sought to examine the expression of E11 in the SCB of two different osteoarthritis animal models, and in samples from patients undergoing total knee replacement (Fig. 1). Immunohistochemical labelling revealed that E11 expression was similar in the SCB of both surgically-induced DMM and non-operated mice in both the lateral and medial aspects of the joint (arrows, Fig. 1A). E11 immunolabelling was also observed in the articular cartilage chondrocytes (Fig. 1A). However, increased E11 expression was observed in the SCB osteocytes in naturally occurring human (Fig. 1 B) and canine (Fig. 1C) osteoarthritis in comparison to unaffected control tissue. This indicates that E11 expression levels are raised in osteoarthritic SCB osteocytes, suggesting that this may be linked to pathology.

### Administration of Bortezomib has no effect on surgically induced osteoarthritis

**Fig. 2:**
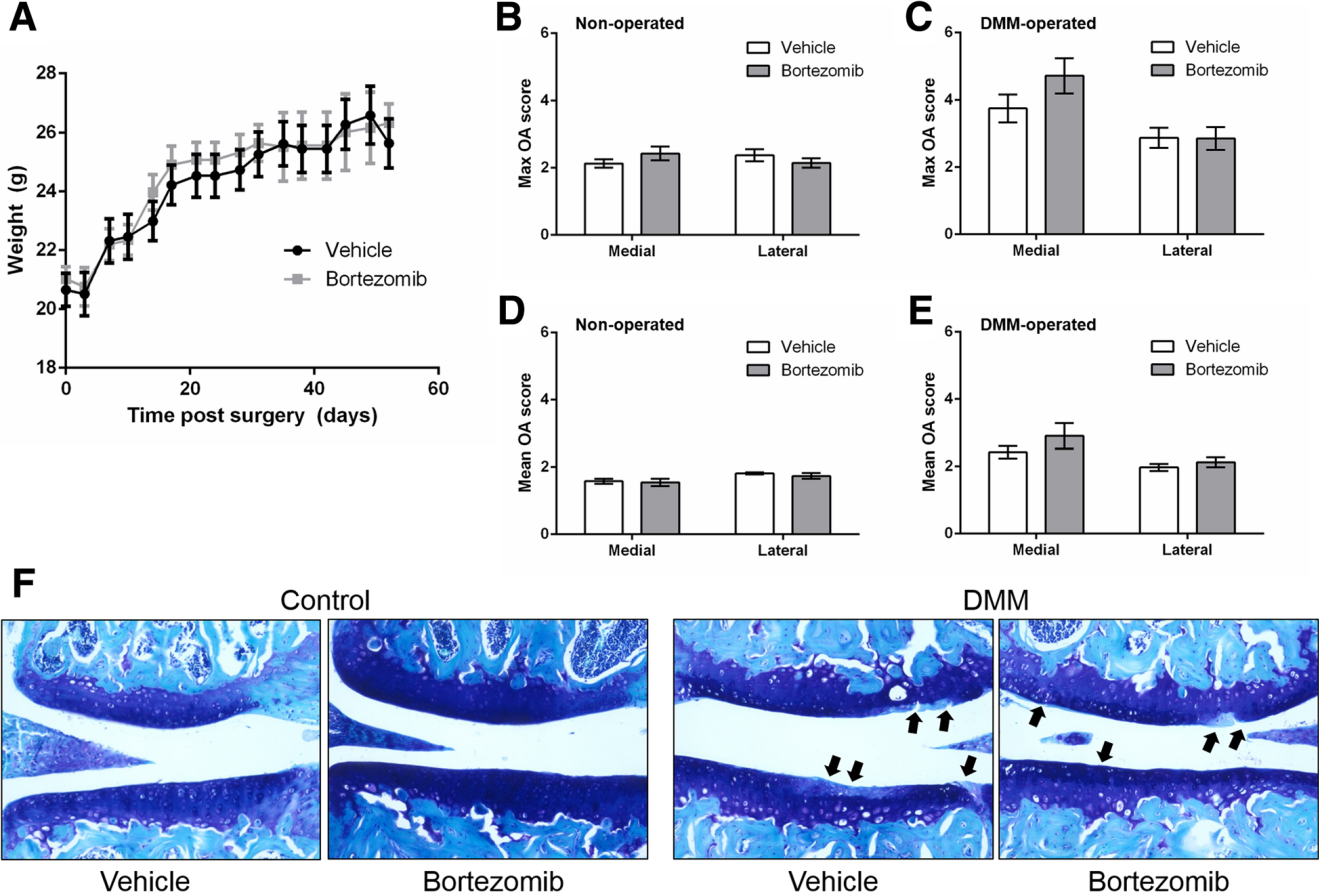
(**a**) Weights of mice treated with Bortezomib or vehicle for days post DMM surgery (**b**) Maximum OARSI score in the medial and lateral compartments of the left (contralateral control) knee joint of Bortezomib and vehicle treated mice (**c**) Maximum OARSI score in the medial and lateral compartments of the right (DMM) knee joint of Bortezomib and vehicle treated mice (**d**) Mean OARSI score in the medial and lateral compartments of the left non-operated (contralateral control) knee joint of Bortezomib and vehicle treated mice (**e**) Mean OARSI score in the medial and lateral compartments of the right (DMM) knee joint of Bortezomib and vehicle treated mice (**f**) Representative histology images of articular cartilage lesions (arrows) in the medial joint compartment. Data are presented as mean ± S.E.M (*n* = 8/group)

We have previously shown that exposure to the proteasome inhibitor, Bortezomib, stabilised E11 expression in vitro [9]*.* Based upon this observation, we hypothesised that Bortezomib treatment would protect against osteoarthritis pathology in vivo through an enhanced stabilisation of E11 and the promotion of osteocyte differentiation. We found no significant differences in the weights of mice treated with Bortezomib in comparison to vehicle-treated mice (Fig. 2 A). There was also no significant difference in either the maximum (Fig. 2 B) or mean (Fig. 2 D) OARSI osteoarthritis scores for the non-operated joints between vehicle and Bortezomib treated mice. The DMM-operated joints showed an expected increase in the OARSI scores in comparison to the non-operated joints (compare Fig. 2C & E to 2B & D) however, there were no significant differences in the OARSI scores between vehicle-treated and Bortezomib-treated joints with DMM (Fig. 2 C, E & F).

**Fig. 3:**
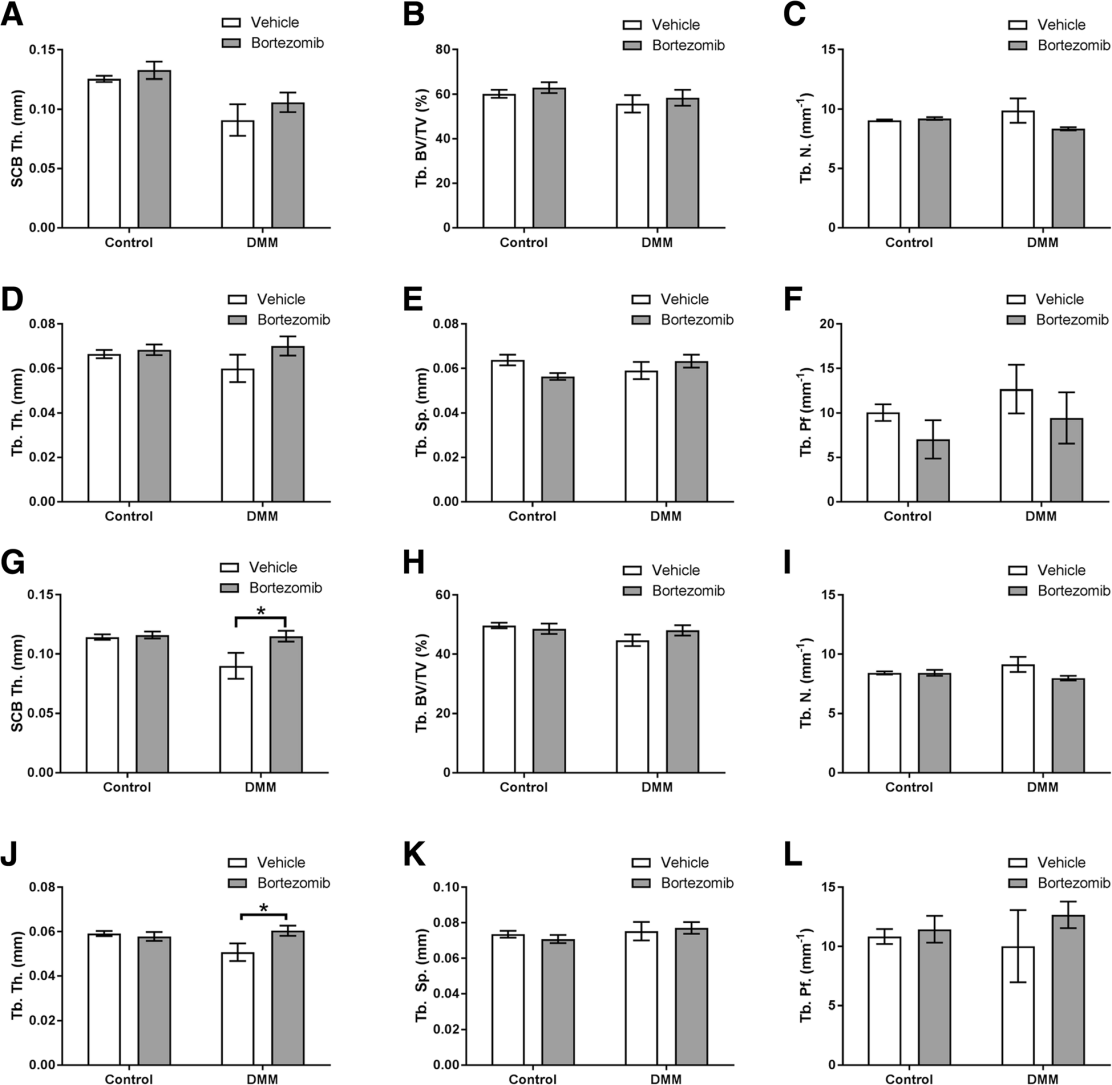
MicroCT analysis of the epiphyseal region of the medial tibia in DMM-operated and non-operated controls (**a**) subchondral bone thickness (SCB Th.) (**b**) trabecular bone volume/tissue volume (Tb. BV/TV) (**c**) trabecular number (Tb. N.) **(d)** trabecular thickness (Tb. Th.) **e ** trabecular separation (Tb. Sp.) **f** trabecular pattern factor (Tb. Pf.). MicroCT analysis of the epiphyseal region of the lateral tibia in DMM-operated and non-operated controls **(g) **subchondral bone thickness (SCB Th.) **(h)** trabecular bone volume/tissue volume (Tb. BV/TV) **(I)** trabecular number (Tb. N.) **(J)** trabecular thickness (Tb. Th.) **(k)** trabecular separation (Tb. Sp.) (l) trabecular pattern factor (Tb. Pf.). Data are presented as mean ± S.E.M (n = 8/group). *P* < 0.05*

In order to determine if there were any SCB abnormalities after DMM surgery in Bortezomib treated mice, we performed microCT analysis. No significant DMM-related differences were observed in the medial tibia SCB plate and epiphyseal trabecular bone parameters (Fig. 3 A–F). In contrast, Bortezomib treated mice exhibited significant DMM-related increases in the SCB thickness (*P* < 0.05, Fig. 3 G) and trabecular thickness (P < 0.05, Fig. 3 J) in the lateral tibia, in comparison to vehicle-treated mice. No significant differences were observed in the other lateral tibia epiphyseal parameters.

**Fig. 4:**
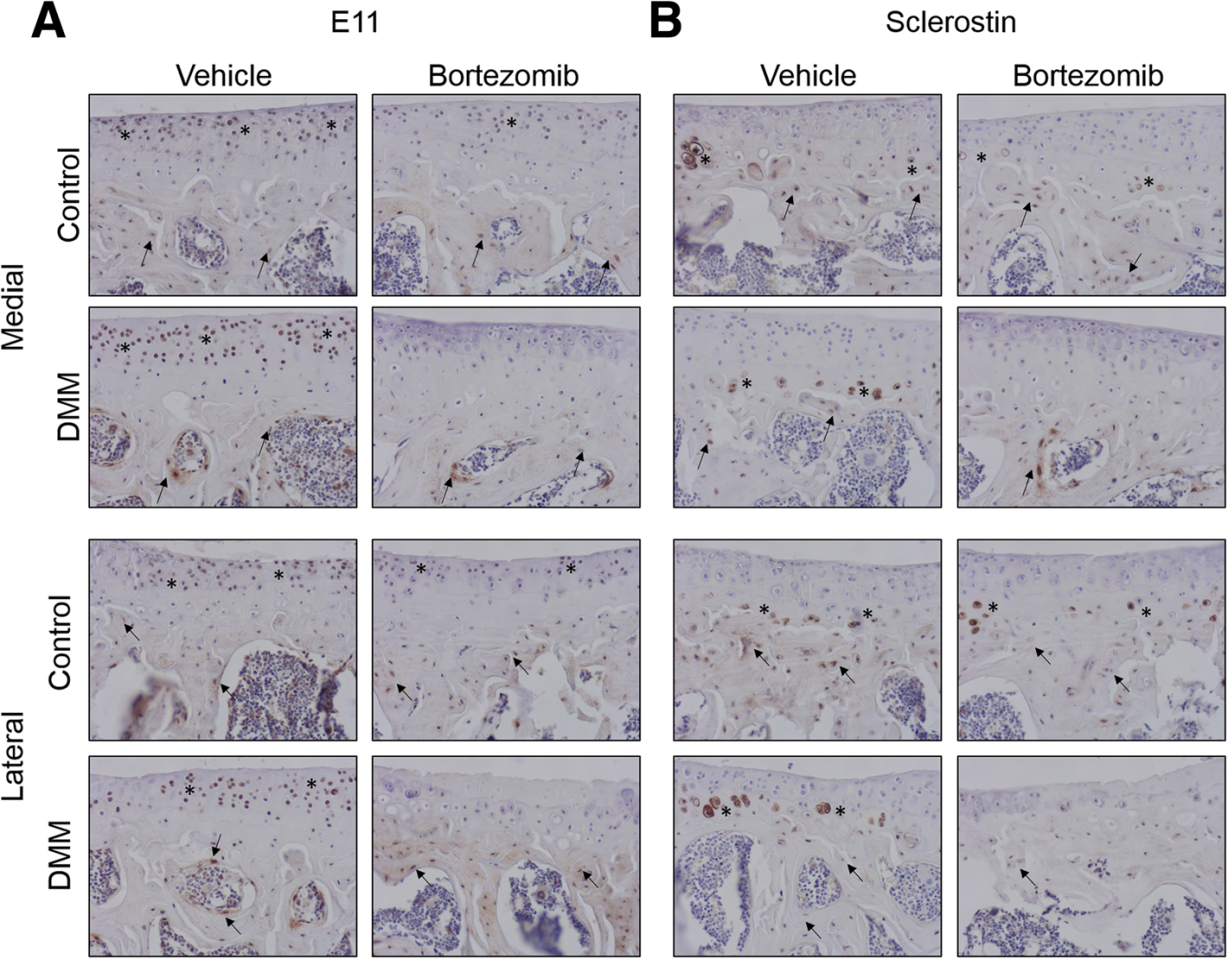
**(a)** E11 immunohistochemistry in the left non-operated (contralateral control) and right (DMM) knee joints of vehicle and Bortezomib treated mice **(b)** sclerostin immunohistochemistry in the left non-operated (contralateral control) and right (DMM) knee joints of vehicle and Bortezomib treated mice. Arrows are representative of E11 positive osteocytes and asterisks indicate positive E11 immunolabelling in the chondrocytes

To assess whether Bortezomib did indeed stabilise E11 expression, we performed immunolabelling for E11 in both non-operated control and DMM-operated knee joints with or without Bortezomib treatment. In the vehicle-treated joints, E11 was expressed in the SCB osteocytes as expected, and also in the chondrocytes of the superficial and middle zones of the articular cartilage (Fig. 4 A). In accordance with our results in Fig. 1 A, DMM had no effect on the SCB expression of E11 (Fig. 4 A). The administration of Bortezomib, however, provoked decreases in the articular cartilage chondrocyte expression of E11, with no apparent differences in the SCB osteocyte expression, in both non-operated and DMM mouse joints (Fig. 4 A).

Immunolabelling for sclerostin showed positive labelling in the hypertrophic chondrocytes and the SCB osteocytes in the non-operated joint of vehicle-treated mice (Fig. 4 B). With DMM, there was no apparent effect on sclerostin expression by SCB osteocytes, however, there was a focal loss of sclerostin expression from the hypertrophic chondrocytes (Fig. 4 B). This loss was more apparent with Bortezomib treatment in both non-operated and DMM mouse joints (Fig. 4 B). These data suggest that the administration of the proteasome inhibitor Bortezomib is unable to protect against surgically induced osteoarthritis pathology, and has no effect on E11 expression in the SCB, but does diminish sclerostin expression in hypertrophic chondrocytes of the articular cartilage.

### Applied loading does not induce articular cartilage lesions or osteophyte formation in E11 cKO mice

As the administration of Bortezomib was unable to modify SCB E11 expression and had no effect on osteoarthritis induced by DMM, we next examined whether the conditional deletion of E11 from bone modifies susceptibility to early adaptive processes and joint vulnerability to osteoarthritis induction by a mechanically-induced post-traumatic osteoarthritis model. We have shown previously that 2 weeks of applied knee joint loading is sufficient to induce localized articular cartilage lesions in the lateral femur [14] and as such, we loaded 12-week-old cKO and WT mice and examined the lateral femur articular cartilage for load-induced lesions.

**Fig. 5:**
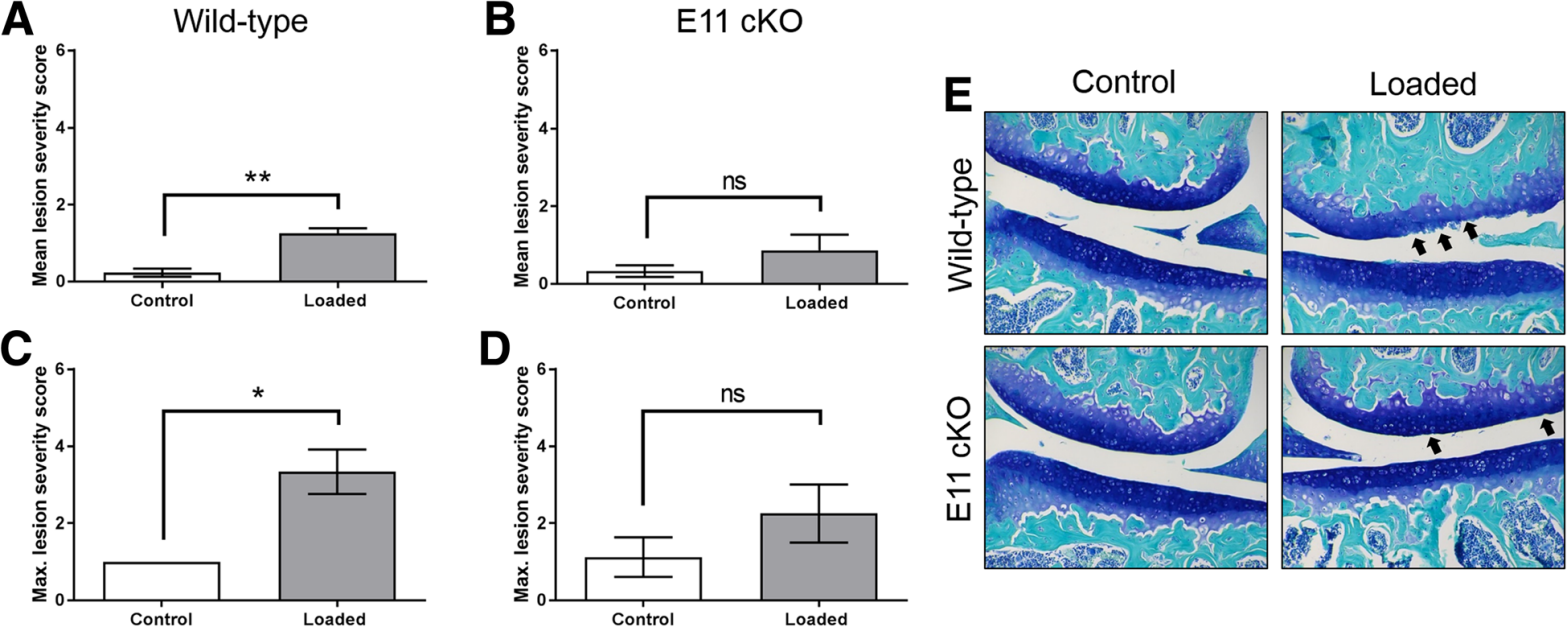
**a** Mean OARSI score in the lateral femur of control and loaded knee joints of WT mice. **b** Mean OARSI score in the lateral femur of control and loaded knee joints of cKO mice. c Maximum OARSI score in the lateral femur of control and loaded knee joints of WT mice. **d** Maximum OARSI score in the lateral femur of control and loaded knee joints of cKO mice. **e** Representative histology images of articular cartilage lesions (arrows) in the lateral femur. Data are presented as mean ± S.E.M (n > 3/group). P < 0.05*; *P* < 0.01**

**Fig. 6:**
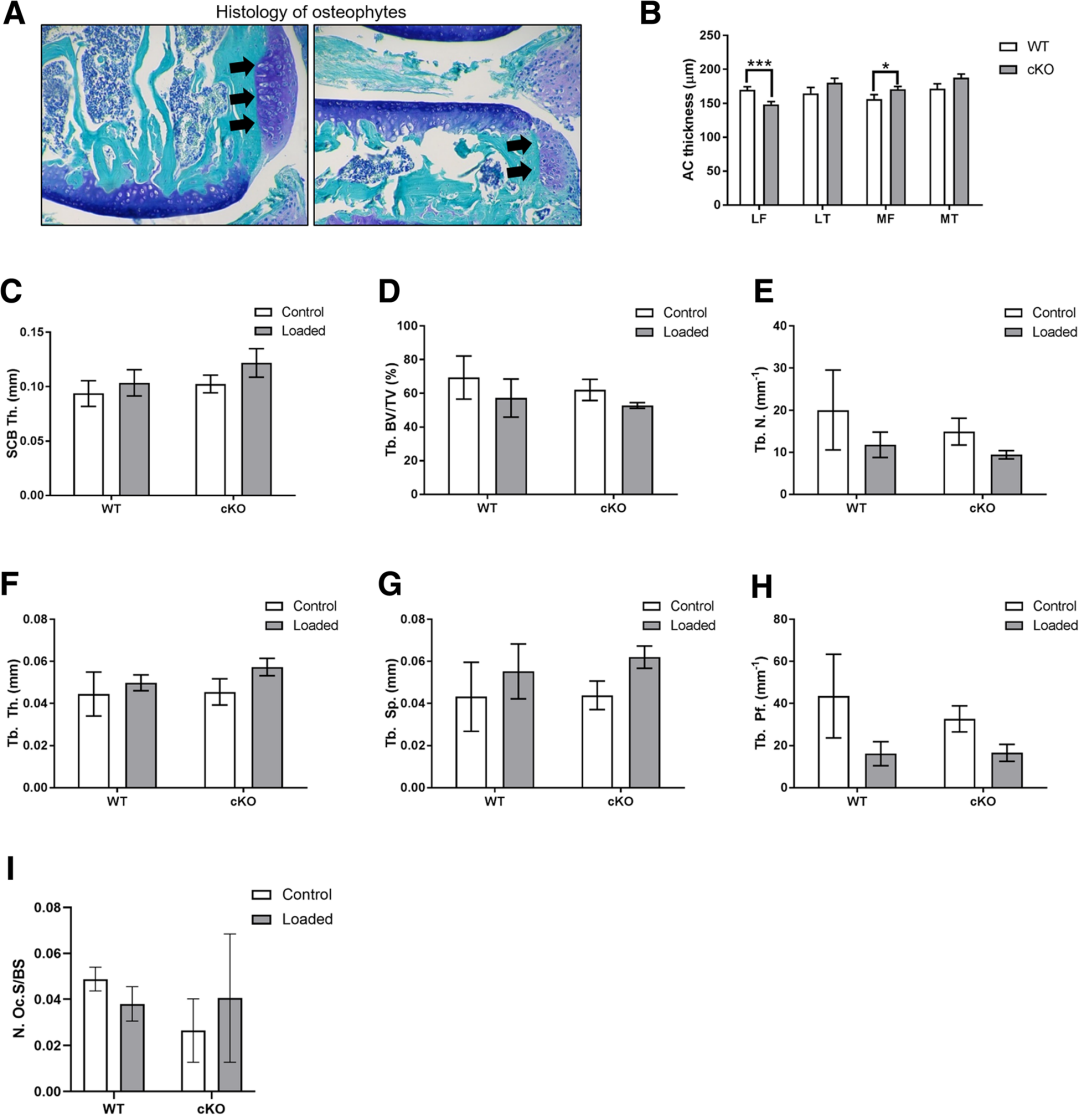
**(a)** Histology images of osteophyte formation (arrows) in loaded joints from WT mice **(b)** Articular cartilage thickness (LF – lateral femur, LT – lateral tibia, MF – medial femur, MT – medial tibia). MicroCT analysis of the epiphyseal region of the lateral femur **(c)** subchondral bone plate thickness (SCB Th.) **(d)** epiphyseal trabecular bone volume/tissue volume (Tb. BV/TV) **(e)** epiphyseal trabecular number (Tb. N.) **(f)** epiphyseal trabecular thickness (Tb. Th.) **(g)** epiphyseal trabecular separation (Tb. Sp.) **(h)** epiphyseal trabecular pattern factor (Tb. Pf.). **i** Number of osteoclasts / bone surface. Data are presented as mean ± S.E.M (n > 3/group). P < 0.05*; *P* < 0.001***

We found that, in concordance with our previous work, WT mice exhibited significant articular cartilage lesions in the lateral femur, in comparison to non-loaded limbs (*p* < 0.01; Fig. 5 A, C, E). However, 12-week-old cKO mice, in contrast, did not develop significant lateral femur lesions following 2 weeks of applied loading, in comparison to non-loaded limbs (Fig. 5 B, D, E). However, no significant differences between loaded cKO and WT limbs were observed. Furthermore, osteophytes were observed by histology as expected in the loaded knee joints of all WT mice but not in loaded knee joints of E11 cKO mice (Fig. 6A & B). To identify baseline differences in articular cartilage structure which may underpin protection against load-induced lesions, we next measured articular cartilage thickness. Surprisingly, we found that the articular cartilage in the lateral femur of our E11 cKO mice (non-loaded) was significantly thinner than the WT mice (*P* < 0.001, Fig. 6 B). This was however counterbalanced by a significant increase in the thickness of the medial femur articular cartilage in the cKO mice (*P* < 0.05, Fig. 6 B). Examination of the lateral femur SCB plate surprisingly revealed no significant differences in the bone plate thickness (Fig. 6 C). Similarly, no significant differences were observed in the femoral epiphyseal trabecular parameters (Fig. 6 D - H). Consistent with this and with our previously published data, no significant differences were observed in osteoclast number per bone surface (Fig. 6 I) [13]. Together, these data suggest that the conditional deletion of E11 from bone protects against articular cartilage lesion and osteophyte induction in response to transient joint loading.

## Discussion

Here we reveal that the bone-specific conditional deletion of E11 in mice is protective against load induced osteoarthritis pathology. This is evidenced by the restriction of both the load-induced development of articular cartilage lesions and osteophyte formation in our E11 cKO mice. We also reveal that Bortezomib fails to exert any protection against osteoarthritis development in a surgical model of osteoarthritis (DMM). This conclusion was unexpected and opposite to our original hypothesis in which we speculated that disruption to the integrity of the osteocyte network would lead to greater osteoarthritis vulnerability.

Osteocytes are the most numerous bone cell type (> 95% of bone cells) and are essential to bone structure and function. They have a unique morphology with long dendritic processes creating multicellular networks permeating the entire bone matrix [3, 21]. Historically considered passive ‘place-holders’, osteocytes have now emerged as versatile orchestrators of bone remodelling as they regulate both osteoblast (bone-forming cells) and osteoclast (bone-resorbing cells) function [22, 23]. In osteoarthritis, osteocytes are known to have a dysfunctional morphology with shorter and fewer dendritic processes [4]. As E11 is essential for the formation of osteocyte dendrites, we therefore hypothesised that the ablation of E11 from bone cells would lead to SCB thickening and exacerbated osteoarthritis pathology via a decreased osteocyte production of the bone formation inhibitor, sclerostin. However, in contrast to our expectations, we observed the opposite outcome. Our loading model used herein is non-invasive and does not induce anterior cruciate ligament rupture, thus avoiding complications that surgical methods have surrounding risk of disturbances to peri-articular tissues and disease progression. Further, whilst DMM relies on permanent, intransient destabilisation where indirect induction and progression of articular cartilage lesions are inseparable, joint loading in our model is controlled and transient, allowing direct induction of lesions and separation from progression. The data from our loading regime (6 loads in 2 weeks) thus allows examination of load-induced early osteoarthritis induction [14]. Our data indicate that less efficient osteocyte differentiation and dendrite formation, due to the hypomorphic deletion of E11, protects against the induction of osteoarthritic articular cartilage in response to transient loading episodes. Further, our data presented here suggests that a disrupted osteocyte morphology occurs in response to osteoarthritis pathology, rather than being causative. This does not however negate the possibility of E11 deficiency increasing vulnerability to other stratifications of osteoarthritis. It is also important to consider the sample size used for our WT mice as a potential limitation of this study. However, using the in vivo loading model, similar small group sizes have been used to resolve statistically significant load-related differences in articular cartilage lesions, surface strains by digital image correlation and osteocyte protein expression by immunolabelling [14, 24, 25]. These results indicate the very high level of reproducibility and experimental robustness of this loading model and provides us with confidence in our interpretation of our data.

Herein we also show that the bone specific deletion of E11 results in reduced articular cartilage thickness in the lateral femur. This is somewhat surprising as it is thought that a thinner articular cartilage is more susceptible to tensile strains, and therefore load induced trauma [26, 27]. Further, as both sclerostin and E11 are expressed in chondrocytes, and as no effects were seen on osteoclast activity or SCB architecture, the protection to load-induced osteoarthritis afforded by E11 deletion in bone may, at least partially, reflect E11’s currently undefined role in the articular cartilage.

We have recently shown using in vitro osteocyte differentiation that late osteoblast E11 protein levels are regulated post-translationally by proteasome degradation and that their preservation, through use of proteasome inhibitors, such as Bortezomib, leads to the induction of an osteocyte-like morphology [9]. Bortezomib is used in vivo for the treatment of multiple myeloma and it is undergoing clinical trials for epithelial cancer treatment [28]. Moreover, it has been shown that Bortezomib prevents the degradation of collagen type II and the induction of MMP13 in vitro, thereby suggesting that it may have therapeutic effects in the context of osteoarthritis [29]. We therefore speculated that administration of the proteasome inhibitor, Bortezomib, in vivo would exert a protection against osteoarthritis development in an alternative surgically-induced model. We found that the administration of 1 mg/kg Bortezomib, via intraperitoneal injection, to mice undergoing DMM surgery had no effect however on osteoarthritis pathology. This concentration and route of delivery has been shown previously to successively reduce proteasome 20S and mitigate histopathological manifestation of pancreatic injury in mice [18]. This is in contrast to a recent publication which showed that the administration of another proteasome inhibitor, MG132, protects against DMM-induced osteoarthritis [30]. There are many possible explanations as to why we observed these contrasting results, the most likely of which is that MG132 and Bortezomib are different types of proteasome inhibitors – MG132 is a peptide aldehyde which also inhibits certain cysteine proteinases, whereas Bortezomib is a peptide boronate inhibitor [31]. Whilst Bortezomib is currently being developed in the clinic, it would be interesting to examine the effects of other proteasome inhibitors within these subcategories to explore whether they exert modification in osteoarthritis development. It is also pertinent to consider our immunohistochemistry results, which showed that the in vivo administration of Bortezomib was not associated with any modification in E11 expression levels in the SCB osteocyte. The failure of in vivo Bortezomib administration to recapitulate its in vitro effects on E11 expression may indeed offer an explanation for lack of effect on osteoarthritis severity. It is nonetheless intriguing that in vivo Bortezomib treatment instead provoked decreased levels of E11 and sclerostin expression in articular cartilage chondrocytes – thus indicating that our Bortezomib dosing procedure was biologically effective in cartilage. The reasons for these observations require further study. It must also be borne in mind that the proteasome has manifold effects on cellular metabolic and signalling pathways, and its effects will not be limited to those we have analysed here.

The data generated here contribute to our understanding of osteoarthritis development and to our pursuit of a disease-modifying treatment. We have shown that the clinically-relevant drug Bortezomib, was not found in this study to have any therapeutic potential in a surgical model of osteoarthritis. We have however shown that the precise control of E11 is crucial in SCB function in osteoarthritis and that the regulatory networks controlling E11 osteocyte expression are more complex in vivo than they are in vitro*.* Furthermore, the data presented here offer further support for the role of cartilage: bone interactions in the development of osteoarthritis.

## Conclusions

Overall, these data suggest that an intact osteocyte network in the SCB contributes to the development of mechanically-driven osteoarthritis. Further, the data presented here indicate that the molecular pathways that preserve the osteocyte network, such as those driven by E11, may be targeted to limit osteoarthritis pathogenesis.

## Additional file

Additional file 1: **Table S1.** Percentage Identity Matrix for E11. (DOCX 13 kb)

## Data Availability

The datasets used and/or analysed during the current study are available from the corresponding author on reasonable request.

## Abbreviations

BV/TV: Bone volume/tissue volume
cKO: Conditional knockout
DMM: Destabilisation of the medial meniscus
LF: Lateral femur
LT: Lateral tibia
MF: Medial femur
microCT: Micro-computed tomography
MT: Medial tibia
ROI: Region of interest
SCB Th: Subchondral bone thickness
SCB: Subchondral bone
Tb. BV/TV: Trabecular bone volume/tissue volume
Tb. N: Trabecular number
Tb. Pf: Trabecular pattern factor
Tb. Sp: Trabecular separation
Tb. Th: Trabecular thickness
WT: Wild-type

## References

[1] Mansell JP, Collins C, Bailey AJ (2007). Bone, not cartilage, should be the major focus in osteoarthritis. Nat Clin Pr Rheumatol.

[2] Karsdal MA, Leeming DJ, Dam EB, Henriksen K, Alexandersen P, Pastoureau P (2008). Should subchondral bone turnover be targeted when treating osteoarthritis?. Osteoarthr Cartil.

[3] Dallas SL, Prideaux M, Bonewald LF (2013). The osteocyte: an endocrine cell ... And more. Endocr Rev.

[4] Jaiprakash A, Prasadam I, Feng JQ, Liu Y, Crawford R, Xiao Y (2012). Phenotypic characterization of osteoarthritic osteocytes from the sclerotic zones: a possible pathological role in subchondral bone sclerosis. Int J Biol Sci.

[5] Wu L, Guo H, Sun K, Zhao X, Ma T, Jin Q (2016). Sclerostin expression in the subchondral bone of patients with knee osteoarthritis. Int J Mol Med.

[6] Zarei A, Hulley PA, Sabokbar A, Javaid MK (2017). Co-expression of DKK-1 and Sclerostin in subchondral bone of the proximal femoral heads from osteoarthritic hips. Calcif Tissue Int.

[7] Zhang K, Barragan-Adjemian C, Ye L, Kotha S, Dallas M, Lu Y (2006). E11/gp38 selective expression in osteocytes: regulation by mechanical strain and role in dendrite elongation. Mol Cell Biol.

[8] Prideaux M, Loveridge N, Pitsillides AA, Farquharson C (2012). Extracellular matrix mineralization promotes E11/gp38 glycoprotein expression and drives osteocytic differentiation. PLoS One.

[9] Staines Katherine A., Prideaux Matt, Allen Steve, Buttle David J., Pitsillides Andrew A., Farquharson Colin (2015). E11/Podoplanin Protein Stabilization Through Inhibition of the Proteasome Promotes Osteocyte Differentiation in Murine in Vitro Models. Journal of Cellular Physiology.

[10] Sprague L, Wetterwald A, Heinzman U, Atkinson MJ (1996). Phenotypic changes following over-expression of sense or antisense E11 cDNA in ROS 17/2.8 cells. J Bone Miner Res.

[11] Martin-Villar E., Megias D., Castel S., Yurrita M. M., Vilaro S., Quintanilla M. (2006). Podoplanin binds ERM proteins to activate RhoA and promote epithelial-mesenchymal transition. Journal of Cell Science.

[12] Martin-Villar E, Borda-d’Agua B, Carrasco-Ramirez P, Renart J, Parsons M, Quintanilla M (2014). Podoplanin mediates ECM degradation by squamous carcinoma cells through control of invadopodia stability. Oncogene..

[13] Staines Katherine A., Javaheri Behzad, Hohenstein Peter, Fleming Robert, Ikpegbu Ekele, Unger Erin, Hopkinson Mark, Buttle David J., Pitsillides Andrew A., Farquharson Colin (2017). Hypomorphic conditional deletion of E11/Podoplanin reveals a role in osteocyte dendrite elongation. Journal of Cellular Physiology.

[14] Poulet B, Hamilton RW, Shefelbine S, Pitsillides AA (2011). Characterizing a novel and adjustable noninvasive murine joint loading model. Arthritis Rheum.

[15] Glasson S.S., Blanchet T.J., Morris E.A. (2007). The surgical destabilization of the medial meniscus (DMM) model of osteoarthritis in the 129/SvEv mouse. Osteoarthritis and Cartilage.

[16] Sophocleous A, Börjesson AE, Salter DM, Ralston SH (2015). The type 2 cannabinoid receptor regulates susceptibility to osteoarthritis in mice. Osteoarthr Cartil.

[17] Inglis JJ, McNamee KE, Chia S-L, Essex D, Feldmann M, Williams RO (2008). Regulation of pain sensitivity in experimental osteoarthritis by the endogenous peripheral opioid system. Arthritis Rheum.

[18] Zhu Q, Lin X, Liu X, Hou T, Zhang M, Wang N (2018). Dynamic changes of proteasome and protective effect of bortezomib, a proteasome inhibitor, in mice with acute pancreatitis. Biochem Biophys Res Commun.

[19] Poulet B, de Souza R, Kent AV, Saxon L, Barker O, Wilson A (2015). Intermittent applied mechanical loading induces subchondral bone thickening that may be intensified locally by contiguous articular cartilage lesions. Osteoarthr Cartil.

[20] Glasson SS, Chambers MG, Van Den Berg WB, Little CB (2010). The OARSI histopathology initiative – recommendations for histological assessments of osteoarthritis in the mouse. Osteoarthr Cartil.

[21] Bonewald LF (2012). The amazing osteocyte. J Bone Min Res.

[22] Xiong J, Onal M, Jilka RL, Weinstein RS, Manolagas SC, O’Brien CA (2011). Matrix-embedded cells control osteoclast formation. Nat Med.

[23] Nakashima T, Takayanagi H (2011). New regulation mechanisms of osteoclast differentiation. Ann N Y Acad Sci.

[24] Javaheri B, Carriero A, Wood M, De Souza R, Lee PD, Shefelbine S (2018). Transient peak-strain matching partially recovers the age-impaired mechanoadaptive cortical bone response. Sci Rep.

[25] Carriero A, Pereira AF, Wilson AJ, Castagno S, Javaheri B, Pitsillides AA (2018). Spatial relationship between bone formation and mechanical stimulus within cortical bone: combining 3D fluorochrome mapping and poroelastic finite element modelling. Bone Reports.

[26] Wilson W, van Burken C, van Donkelaar C, Buma P, van Rietbergen B, Huiskes R (2006). Causes of mechanically induced collagen damage in articular cartilage. J Orthop Res.

[27] Poulet B, Westerhof TA, Hamilton RW, Shefelbine SJ, Pitsillides AA (2013). Spontaneous osteoarthritis in Str/ort mice is unlikely due to greater vulnerability to mechanical trauma. Osteoarthr Cartil.

[28] Ria R, Reale A, Vacca A (2014). Novel agents and new therapeutic approaches for treatment of multiple myeloma. World J Methodol.

[29] Hu W, Zhang W, Li F, Guo F, Chen A (2014). Bortezomib prevents the expression of MMP-13 and the degradation of collagen type 2 in human chondrocytes. Biochem Biophys Res Commun.

[30] Radwan M, Wilkinson DJ, Hui W, Destrument AP, Charlton SH, Barter MJ (2015). Protection against murine osteoarthritis by inhibition of the 26S proteasome and lysine-48 linked ubiquitination. Ann Rheum Dis.

[31] Kisselev AF, van der Linden WA, Overkleeft HS (2012). Proteasome inhibitors: an expanding Army attacking a unique target. Chem Biol.

