# Supplementary material for: Conditional deletion of E11/podoplanin in bone protects against load-induced osteoarthritis
Source: BMC Musculoskelet Disord. 2019 Jul 27;20:344. doi: 10.1186/s12891-019-2731-9 (PMC6661085; doi:10.1186/s12891-019-2731-9)
Supplement: Supplementary file 1 — Table S1. Percentage Identity Matrix for E11. (DOCX 13 kb) [file 12891_2019_2731_MOESM1_ESM.docx]

|  | **Mouse** | **Human** | **Canine** |
| --- | --- | --- | --- |
| **Mouse** | 100 | 47.53 | 53.70 |
| **Human** | 47.53 | 100 | 64.81 |
| **Canine** | 53.70 | 64.81 | 100 |

**Additional file 1: Table S1.** Percentage Identity Matrix for E11
